# Interictal and seizure‐onset scalp electroencephalographic patterns in malformations of cortical development

**DOI:** 10.1002/epi.70193

**Published:** 2026-03-14

**Authors:** Lubna Shakhatreh, Liz Edenberg P. Quiles, Zhibin Chen, Ofer M. Gonen, Saba Mohidat, Lyn Millist, Callum Hollis, Andrea Sundram, Piero Perucca, Terence J. O'Brien, Patrick Kwan

**Affiliations:** ^1^ Department of Neuroscience School of Translational Medicine, Monash University Melbourne Victoria Australia; ^2^ Department of Neurology Royal Melbourne Hospital Melbourne Victoria Australia; ^3^ Department of Neurology Alfred Health Melbourne Victoria Australia; ^4^ Department of Neurology Monash Medical Centre Clayton Victoria Australia; ^5^ Department of Neurology Institute of the Neurological Sciences Medical City Pasig City Philippines; ^6^ School of Public Health and Preventive Medicine Monash University Melbourne Victoria Australia; ^7^ Nucleus Network Melbourne Victoria Australia; ^8^ Department of Neurology Al‐Balqa Applied University As‐Salt Jordan; ^9^ Bladin‐Berkovic Comprehensive Epilepsy Program, Department of Neurology Austin Health Melbourne Victoria Australia; ^10^ Department of Medicine (Austin Health) Epilepsy Research Centre, University of Melbourne Melbourne Victoria Australia; ^11^ Department of Medicine (Royal Melbourne Hospital) University of Melbourne Melbourne Victoria Australia

**Keywords:** epilepsy, interictal patterns, malformations of cortical development, scalp EEG, seizure‐onset pattern

## Abstract

**Objective:**

Malformations of cortical development (MCDs) are a frequent cause of drug‐resistant epilepsy and a common indication for resective epilepsy surgery. As magnetic resonance imaging (MRI) lacks sensitivity for subtle MCDs, supplemental diagnostic tools are needed. This study aimed to characterize scalp electroencephalographic (EEG) patterns in MCDs and investigate their association with surgical outcomes.

**Methods:**

This was a retrospective case–control study including patients who underwent inpatient video‐EEG monitoring at two Australian hospitals. Cases were individuals with MCDs confirmed on MRI or histopathology; controls included patients with other focal epilepsies. Two epileptologists independently reviewed interictal and seizure‐onset EEG patterns using a standardized classification framework. Patterns were compared between cases and controls and assessed with respect to postoperative seizure outcomes, adjusting for antiseizure medication reduction.

**Results:**

We included 38 cases with MCDs (52.6% females, median age = 34 years) and 114 controls (45.6% females, median age = 41 years). Among interictal patterns, repetitive epileptiform discharges type 1 and type 2 were more prevalent in patients with MCDs than controls (*p* = .002 and .005, respectively). Focal fast epileptiform discharges were also more frequent in MCD patients, after excluding nonlesional focal epilepsy controls (*p* = .038). Among seizure‐onset patterns, paroxysmal fast activity was more prevalent in MCDs (*p* < .001). Among 38 patients who underwent surgery, 70.0% of MCDs and 75.0% of controls had favorable outcomes. No EEG pattern predicted postoperative seizure outcomes.

**Significance:**

Distinct scalp EEG patterns may support differentiation of MCDs from other focal epilepsies. Larger prospective studies are needed to clarify their role in detecting MRI‐negative MCD or guiding targeted imaging.

**Key points**

- Repetitive epileptiform discharges and focal fast discharges on scalp EEG were more frequent in MCD than other focal epilepsies.
- Paroxysmal fast activity was the seizure‐onset pattern most strongly linked with MCD.
- Scalp EEG patterns may support differentiation of MCD from other focal epilepsies.
- No interictal or seizure‐onset scalp EEG pattern predicted postsurgical seizure outcomes.

## 1 INTRODUCTION

Malformations of cortical development (MCDs) comprise a heterogeneous spectrum of cerebral abnormalities resulting from a focal or regional distortion in normal cerebral development. MCDs are frequently associated with epilepsy, which tends to be drug‐resistant, often requiring surgical intervention for seizure control. Although magnetic resonance imaging (MRI) is the gold standard for noninvasive diagnosis of MCDs, its sensitivity remains suboptimal for subtle lesions such as some focal cortical dysplasia (FCD) and polymicrogyria (PMG). For instance, histopathology analysis reveals FCD in 42%–73% of MRI‐negative drug‐resistant epilepsy (DRE) patients undergoing epilepsy surgery, highlighting the limitations of conventional imaging modalities and emphasizing the need for complementary diagnostic tools.^1^, ^2^, ^3^

Several intracranial interictal and seizure‐onset electroencephalographic (EEG) patterns have been described in MCDs. The first described patterns were (semi‐)continuous rhythmic spiking and repetitive bursts in intraoperative electrocorticographic recording of patients with FCDs.^4^ These patterns have played an essential role in delineating the extent of lesional tissue to guide resective epilepsy surgery, improving postoperative seizure outcomes.^4^ Subsequent studies have reported additional intracranial patterns in FCDs and other MCD types such as low‐voltage fast activity, burst of polyspikes, and delta brush.^5^, ^6^, ^7^, ^8^

Scalp EEG is a noninvasive, widely accessible tool that forms a critical part of presurgical evaluation.^9^ Specific scalp EEG patterns such as “brushes” and repetitive epileptiform discharges (RED) type 1 and type 2, have been observed in FCDs and proposed as potential biomarkers.^10^, ^11^ However, studies investigating scalp EEG patterns across the broader spectrum of MCDs remain limited,^11^, ^12^ leaving the diagnostic value of scalp EEG in MCDs insufficiently explored.

Resective epilepsy surgery is considered the best treatment option for achieving seizure freedom in eligible patients with DRE. Several predictors of postoperative seizure outcomes have been described, including complete resection of the epileptogenic lesion.^13^ Besides complete resection, specific intracranial EEG patterns have also been proposed as potential predictors of postoperative seizure outcomes.^8^, ^14^ However, the predictive value of scalp EEG in this context remains underexplored and warrants further investigations.

In this study, we primarily aimed to characterize interictal and seizure‐onset scalp EEG patterns in patients with drug‐resistant focal epilepsy due to MCDs, in comparison to patients with other or no identifiable pathology. A secondary, exploratory objective was to examine whether certain scalp EEG patterns can predict postoperative outcomes in individuals who underwent resective epilepsy surgery.

## 2 MATERIALS AND METHODS

### 2.1 Study design and subjects

This was a case–control study. We retrospectively reviewed scalp EEG recordings for patients with focal epilepsy admitted to the video‐EEG monitoring (VEM) unit at the Royal Melbourne Hospital and the Alfred Hospital, Australia between 2000 and 2021. We included patients with focal epilepsy and available EEG data. Patients with generalized epilepsy, combined focal and generalized epilepsy, or unavailable EEG data were excluded. We defined as “cases” those patients whose brain MRI or histopathology demonstrated MCD (mild MCD, FCD, PMG, heterotopia, schizencephaly, tuberous sclerosis complex [TSC], and complex MCD [more than one type]) as a cause of their epilepsy. MCD types were classified according to their radiological or histopathological appearances, based on the Barkovich et al. 2012 classification system.^15^ To identify EEG correlates specific to MCDs, cases were compared to controls, who were patients with other etiologies of focal epilepsy: (1) temporal lobe epilepsy associated with hippocampal sclerosis (HS); (2) acquired focal epilepsy (i.e., epilepsy secondary to stroke, vascular malformation, cerebral tumor, or traumatic brain injury); or (3) nonlesional focal epilepsy (i.e., no epileptogenic lesion detected on MRI or histopathology). This heterogenous control group was selected to assess the specificity of MCD‐associated EEG patterns across a clinically representative spectrum of focal epilepsies. Patients with radiological or histopathological evidence of MCD were excluded from the control group.

We collected the following clinical data: sex, age at epilepsy onset, age at VEM admission, number of antiseizure medications (ASMs) prescribed at time of admission, changes in ASMs during admission, and frequency of convulsive (i.e., focal to bilateral tonic–clonic seizures) and nonconvulsive seizures at admission (Table S1).^16^ For patients who underwent epilepsy surgery, postoperative seizure outcomes were assessed at the last follow‐up and categorized using Engel's classification.^17^ For patients who were followed for at least 1 year, we categorized postoperative seizure outcomes as either “favorable” or “unfavorable.” The former included outcomes consistent with Engel class I; Engel classes II–IV were regarded as “unfavorable.” Seizures occurring within the first week after surgery were disregarded in the categorization of seizure outcomes.^18^

### 2.2 Standard protocol approvals, registrations, and patient consents

The study was approved by the Alfred Hospital Ethics Committee and the Royal Melbourne Hospital QA Ethics and registered with the Monash University Human Research Ethics Committee (Project ID: 49006). Patient consent was not required due to the low‐risk nature of the project.

### 2.3 MRI acquisition

All patients had brain MRI with spin‐echo T1‐weighted, T2‐weighted, and fluid‐attenuated inversion recovery sequences. Axial and coronal sections were used to assess mesial temporal structures. Until 2005, MRI studies were carried out on a Genesis Signa 1.5 T (GE Medical Systems); thereafter, the scans were performed on a Magnetom Avanto 1.5 T and a Magnetom Trio Tim 3 T (Siemens Medical Solutions).^19^

### 2.4 Scalp EEG acquisition

Scalp EEGs were recorded using the international 10–20 system. Patients who were admitted for surgical evaluation had additional inferotemporal electrodes from the 10–10 system (F9, FT9, T9, TP9, P9, F10, T10, FT10, TP10, and P10) with or without other closely spaced electrodes. The EEGs were acquired using the Compumedics Profusion system with a default sampling rate of 250 Hz. Each study had a ground and reference applied located in an electrically quiet position on the head, generally adjacent to the Cz position. High‐frequency filter and time constant were configurable but defaulted to 70 Hz and .5 s, respectively. A separate 50‐Hz notch filter was applied if noise minimization was required.

### 2.5 Scalp EEG patterns classification

Two epileptologists (L.S. and L.E.P.Q.) independently reviewed EEG data. Both reviewers were blinded to the patient's clinical data. Initially, they classified the existing annotations and reports to verify comprehensive documentation of all epileptiform findings. In cases of incomplete annotation, they reviewed the entire EEG recording. EEG patterns were classified using a classification framework that comprises the most commonly used pattern designations in the literature, which encompass or match other descriptions in terms of different EEG characteristics such as activity frequency, amplitude, and duration (Table S1).^20^ The framework consists of the following patterns: five interictal patterns (RED type 1,^10^ RED type 2,^10^ brushes,^21^ focal fast epileptiform discharges,^11^ and isolated discharges^11^), and five seizure‐onset patterns (paroxysmal fast activity,^12^ rhythmic slow activity,^12^ repetitive epileptiform discharges,^12^ focal fast wave,^22^ and suppression^12^). For each seizure, the seizure‐onset EEG pattern was determined based on the first 5 s of the initial unequivocal EEG change.

### 2.6 Interrater reliability

We randomly selected approximately 20% of the EEG records of the cases and controls to assess interrater reliability using all annotated epileptiform discharges and recorded seizures. Both reviewers (L.S. and L.E.P.Q.) independently classified the patterns, and interrater reliability was assessed using the intraclass correlation coefficient (ICC) with a corresponding 95% confidence interval (CI) to evaluate the consistency of classifying EEG patterns between the two reviewers. ICC values below .5 suggest inadequate reliability, whereas values ranging from .5 to .75 indicate moderate reliability. Values between .75 and .9 indicate good reliability, and values above .9 reflect excellent reliability.^23^

Following demonstration of satisfactory reliability, the remaining EEG records were divided between the two reviewers. For the initial EEG records used in the interrater reliability testing, any discrepancies in classification were resolved by adopting the interpretation of the reviewer who analyzed the larger proportion of EEGs overall (L.S.).

### 2.7 Statistical analysis

Descriptive statistics were used to summarize the clinical features of individual patient data. Continuous variables were expressed as median and interquartile range (IQR), as they followed nonnormal distributions. Categorical variables were reported as frequency count and percentage. The presence of interictal and seizure‐onset EEG patterns was summarized as frequency counts and percentages.

Mann–Whitney *U*‐tests or chi‐squared tests were performed, as appropriate, to assess differences in demographic and clinical characteristics between MCD cases and controls (with and without nonlesional controls). For group comparison, Fisher exact test was employed to assess differences in the presence of individual interictal and seizure‐onset EEG patterns between patients with MCD and controls before and after excluding the nonlesional focal epilepsy subgroup. This exclusion was performed to account for potential MRI‐undetected MCD in the nonlesional focal epilepsy subgroup that could confound the results.

Multivariable analysis utilized penalized maximum likelihood logistic regression to estimate adjusted odds ratios for interictal and seizure‐onset EEG patterns, preventing bias from the small samples. Generalized linear model with Poisson family, log link, and robust variance estimator was used to calculate the ratio of means for seizure‐onset pattern frequencies. Both analyses were adjusted for ASM withdrawal during VEM.

Penalized maximum likelihood logistic regression was also used to investigate the associations between interictal and seizure‐onset EEG patterns and epilepsy surgery outcomes, adjusted for the diagnostic groups.

The Holm–Bonferroni method was applied separately to correct for multiple comparisons, controlling for familywise errors in the presence of interictal patterns, presence of seizure‐onset EEG patterns, and seizure‐onset pattern frequency, respectively. A *p*‐value < .05 was considered statistically significant. All statistical analyses were performed in Stata version 16.1 (StataCorp).

## 3 RESULTS

### 3.1 Clinical characteristics

A total of 38 cases with MCDs and 114 controls were included (Table 1). Overall, cases were significantly younger than controls in both age at seizure onset and age at VEM admission (*p*‐value = .009 and .017, respectively; Table S2). At the time of VEM, cases were on average taking one more ASM compared to controls (*p*‐value = .034).

**TABLE 1:** Clinical and VEM characteristics for cases and controls.

Clinical and VEM characteristics	Cases, *n* = 38	Controls, *n* = 114		
		HS, *n* = 34	Acquired epilepsy, *n* = 38	Nonlesional epilepsy, *n* = 42
Female sex, *n* (%)	20 (52.6%)	18 (52.9%)	13 (34.2%)	21 (50%)
Median age at seizure onset, years (IQR)	15 (7–20)	19 (9–31)	25 (14–41)	18 (12–27.5)
Median age at VEM admission, years (IQR)	34 (25.8–41)	44 (30.5–54.5)	43 (32.8–57.3)	37 (28–45.8)
Median number of ASMs at time of VEM admission (IQR)	3 (2–3)	2 (2–3)	2 (2–3)	2 (2–3)
Changes in ASMs made during VEM, *n* (%)	28 (73.7%)	30 (88.2%)	32 (84.2%)^a^	36 (85.7%)^b^
Number of VEM days, range	2–8	5–7	4–5	3–7
Median nonconvulsive seizure frequency score at time of VEM admission (range)	8 (2–8)	7 (6–8)	7 (5–8)	7 (4–8)
Median convulsive seizure frequency score at time of VEM admission (range)	2 (2–7)	2 (2–7)	2 (2–5)	2 (2–7)

Abbreviations: ASM, antiseizure medication; HS, hippocampal sclerosis; IQR, interquartile range; VEM, video‐electroencephalographic monitoring.

^a^ Changes made to ASMs during VEM were unknown in one control with acquired epilepsy.

^b^ Changes made to ASMs during VEM were unknown in two controls with nonlesional epilepsy.

Among cases, 20 (52.6%) were female. Fourteen (36.8%) patients had FCD, 11 (28.9%) had heterotopia, five (13.8%) had PMG, four had complex MCD, three had TSC, and one had mild MCD. All were detected on MRI, except for six cases (15.8%) whose MCD was detected on histopathology. The median age of seizure onset was 15 years (IQR = 7–20 years). The median age at VEM admission was 34 years (IQR = 25.8–41 years), with 29 patients (76.3%) having 5 days of EEG monitoring (range = 2–8 days). The median number of ASMs was 3 (IQR = 2–3), with 10 patients having no change made in their ASMs during VEM. The median nonconvulsive seizure frequency score and convulsive seizure frequency score were 8 (IQR = 2–8) and 2 (IQR = 2–7), respectively.

Among controls, 52 (45.6%) were female. Thirty‐four (29.8%) had HS, 38 (33.3%) were diagnosed with acquired epilepsy, and 42 (36.8%) with nonlesional epilepsy. Among all controls, the median age of seizure onset was 21 years (IQR = 11–32.3 years). The median age at VEM admission was 41 years (IQR = 30–52 years), with 103 patients (90.4%) having 5 days of EEG monitoring (range = 3–7 days). The median number of ASMs was 2 (IQR = 2–3), with 13 patients having no change made in their ASMs during VEM. The median nonconvulsive seizure frequency score and convulsive seizure frequency score were 7 (IQR = 5–8) and 2 (IQR = 2–7), respectively. Clinical and VEM characteristics for each subgroup are provided in Table 1.

### 3.2 Interrater reliability

Interrater reliability was assessed on 34 randomly selected EEG records (Table S3). Among interictal patterns, interrater reliability was excellent for isolated discharges (ICC = .98, 95% CI = .93–.98) and good for RED type 1 (ICC = .75, 95% CI = .56–.87). Agreement was poor to negligible for RED type 2 (ICC = −.01, 95% CI = −.35 to .33), focal fast epileptiform discharges (ICC = .00, 95% CI = −.32 to .33), and brushes (ICC = .00, 95% CI = −.33 to .33), likely due to the low number of observations for these patterns.

There was high agreement in the total number of seizures observed (ICC = .96, 95% CI = .92–.98; Table S3). Among seizure patterns, there was good to excellent agreement for paroxysmal fast activity (ICC = .94, 95% CI = .89–.97), rhythmic slow activity (ICC = .92, 95% CI = .85–.96), and repetitive epileptiform discharges (ICC = .98, 95% CI = .97–.99). Agreement was poor to negligible for suppression (ICC = −.04, 95% CI = −.34 to .28), likely due to the low number of observations for this pattern.

### 3.3 Interictal EEG patterns

**FIGURE 1:**
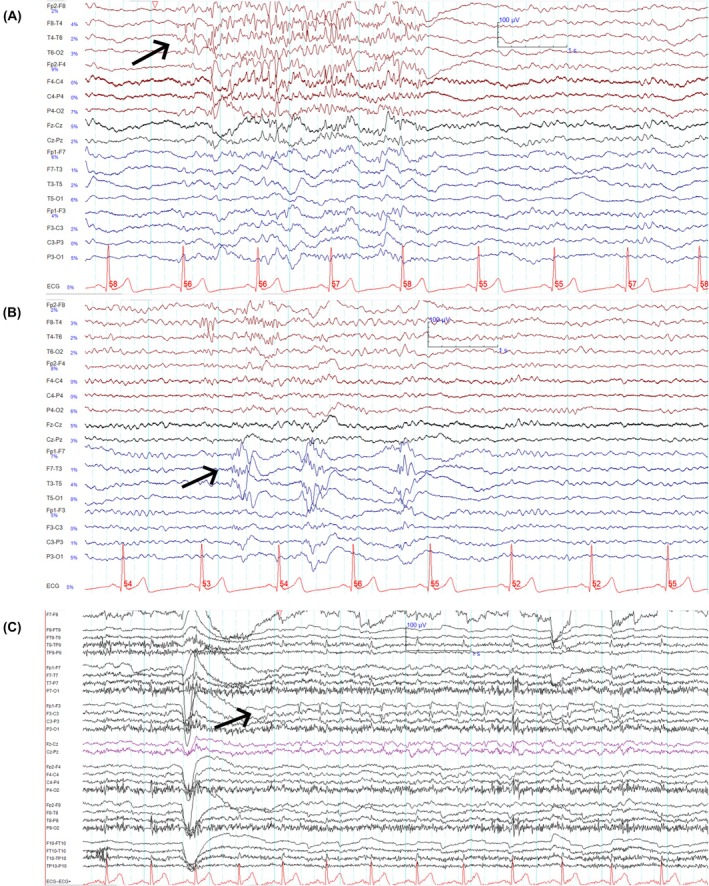
A bipolar montage of scalp electroencephalographic tracings showing (A) fast epileptiform discharges and (B) repetitive epileptiform discharges (RED) type 1 in a patient with bitemporal lobe epilepsy secondary to complex malformation of cortical development, and (C) RED type 2 in a patient with left frontal lobe epilepsy secondary to focal cortical dysplasia type IIb. Arrows indicate the start of the pattern.

Five cases (13.2%) had no interictal epileptiform findings (Table 2). Fifteen cases (39.5%) had more than one interictal pattern. Isolated discharges (81.6%) were the most frequent pattern. RED type 1 and RED type 2 (Figure 1A,B) were identified in 31.6% and 26.3% of cases, respectively. Focal fast epileptiform discharges (Figure 1C) were identified in 10.5% of cases. Brushes were not observed in the MCD group.

**TABLE 2:** Interictal and seizure‐onset electroencephalographic patterns across MCDs and controls.

	MCDs	Controls				MCDs versus controls		MCDs versus controls (excluding nonlesional)	
		HS	Acquired epilepsy	Nonlesional	Subtotal				
						*p*	HB‐corrected *p*	*p*	HB‐corrected *p*
Total *N*	38	34	38	42	114				
Presence of interictal patterns, *n* (%)									
Focal fast epileptiform discharges	4 (10.5)	0 (0)	0 (0)	2 (4.8)	2 (1.8)	.035	.14	.013	.038
Isolated discharges	31 (81.6)	26 (76.5)	31 (81.5)	32 (76.2)	89 (78.1)	.82	1.00	.81	.81
RED type 1	12 (31.6)	1 (2.9)	3 (7.9)	4 (9.5)	8 (7.0)	<.001	.002	<.001	.002
RED type 2	10 (26.3)	1 (2.9)	3 (7.9)	4 (9.5)	8 (7.0)	.003	.015	.005	.019
Brushes	0 (0)	0 (0)	0 (0)	1 (2.4)	1 (.01)	1.00	1.00	N/A	
Presence of seizure‐onset patterns, *n* (%)									
Paroxysmal fast activity	13 (34.2)	1 (2.9)	2 (5.3)	2 (4.8)	5 (4.4)	<.001	<.001	<.001	<.001
Repetitive epileptiform discharges	17 (44.7)	7 (20.6)	9 (23.7)	14 (33.3)	30 (26.3)	.043	.17	.017	.069
Rhythmic slow activity	18 (47.4)	22 (64.7)	18 (47.4)	22 (52.4)	62 (54.4)	.461	1.00	.430	1.00
Suppression	1 (2.6)	2 (5.9)	2 (5.3)	2 (4.8)	6 (5.3)	.681	1.00	.657	.66

Abbreviations: HB, Holm–Bonferroni; HS, hippocampal sclerosis; MCD, malformation of cortical development; N/A, not applicable; RED, repetitive epileptiform discharges.

**FIGURE 2:**
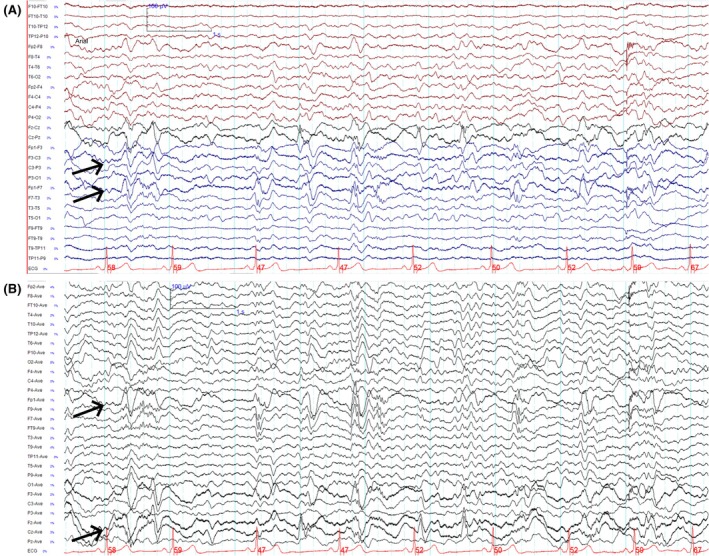
Scalp electroencephalographic tracings showing brush pattern over the left frontocentral region in a patient with nonlesional left frontal epilepsy. (A) Bipolar montage, (B) average montage. Arrows indicate the start of the pattern.

Twenty‐three (20.2%) controls had no interictal epileptiform findings (Table 2). Five patients with HS, five with acquired epilepsy, and eight with nonlesional epilepsy had more than one interictal pattern. Isolated discharges (78.1%) were the most frequent pattern. RED type 1 and RED type 2 were identified in 7% of controls (Table 2). In the nonlesional focal epilepsy subgroup, focal fast epileptiform discharges were identified in two patients (4.8%), whereas brushes were noted in one patient (2.4%; Figure 2).

Across all interictal EEG patterns, RED type 1 and RED type 2 were more frequent in cases compared to controls (*p*‐value = .002 and .015, respectively; Table 2). Focal fast epileptiform discharges were more frequent in cases compared to controls after excluding the nonlesional subgroup (*p*‐value = .038). After adjusting for ASM reduction during the VEM (Table S4), RED type 1 and RED type 2 remained significantly associated with MCDs.

### 3.4 Seizure‐onset patterns

**FIGURE 3:**
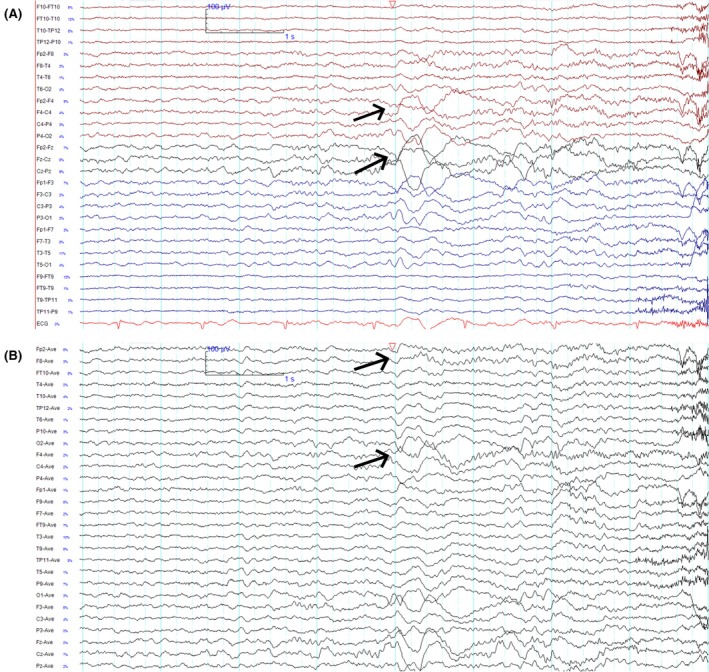
Scalp electroencephalographic tracings showing seizure onset with paroxysmal fast activity over the right frontocentral region (F4, C4, Fz, and Cz) in a patient with right frontal epilepsy secondary to focal cortical dysplasia type IIa. (A) Bipolar montage; (B) average montage. Arrows indicate the start of the pattern.

Among cases, 32 (84.2%) had seizures and six (15.8%) did not have seizures during their VEM admission. Among those who had seizures, 17 (53.1%) had more than one seizure‐onset pattern. A total of 405 seizures were recorded. Twenty seizures (4.9%) demonstrated no clear EEG changes or were contaminated by muscle or movement artifacts. Rhythmic slow activity (47.4%) was the most frequent seizure‐onset pattern, followed by repetitive epileptiform discharges (44.7%) and paroxysmal fast activity (34.2%; Figure 3). Suppression (2.6%) was only seen in one patient (Table 2).

Among controls, 94 (82.5%) had seizures and 20 (17.5%) did not have seizures during their VEM admission. Among those who had seizures, 17 (18.1%) had more than one seizure‐onset pattern. A total of 594 seizures were assessed. One hundred three seizures (17.3%) had no clear EEG changes or were associated with artifact. Rhythmic slow activity (54.4%) was the most frequent seizure‐onset pattern, followed by repetitive epileptiform discharges (26.3%; Table 2). Suppression and paroxysmal fast activity were seen in 5.3% and 4.4%, respectively.

By Fisher exact test (Table 2), paroxysmal fast activity was more frequent in cases compared to controls (*p*‐value < .001). This difference remained statistically significant after adjusting for ASM reduction during the VEM admission (Table S4).

### 3.5 Interictal and seizure‐onset EEG patterns among MCD types

Due to the small sample sizes within each MCD type, formal statistical analyses to assess the associations between individual EEG patterns and specific MCD types were not performed. Overall, isolated discharges were the most common interictal patterns across all MCD types. RED type 1 and RED type 2 were more frequent in FCD (36% and 29%, respectively), whereas RED type 2 was not recognized in polymicrogyria and mild MCD. Focal fast epileptiform discharges were not observed in heterotopia.

Among seizure‐onset patterns, repetitive epileptiform discharges were observed in all MCD types, most frequently in FCDs (57%). Rhythmic slow activity was more frequent in heterotopia (64%). Paroxysmal fast activity was more frequent in PMG (70%) and FCD (60%) but absent in complex MCD and mild MCD cases. Suppression was observed in one patient (7.1%) with FCD.

### 3.6 Postsurgical seizure outcome and EEG patterns

Ten cases and 34 controls underwent resective epilepsy surgery. Two controls were lost to follow‐up, and four were followed for less than 1 year following surgery. Median postoperative follow‐up period was 3 years (IQR = 2.2–4.0 years) among cases and 4.1 years (IQR = 2–5.2) among controls. Seven cases (70%) and 21 controls (75%) achieved favorable postoperative seizure outcomes (Table S5).

One case and one control were excluded from the analysis because VEM was performed after epilepsy surgery. There was no association between postoperative seizure outcomes and diagnostic groups (*p*‐value = .65; Table S5). Thus, the associations between EEG patterns and surgical outcome were not adjusted for diagnosis. The presence of RED type 1 and RED type 2 might be associated with unfavorable postoperative seizure outcomes; however, these effects were not significant after accounting for multiple comparisons (Table 3).

**TABLE 3:** Associations between interictal and seizure‐onset electroencephalographic patterns and epilepsy surgery outcomes.

	OR	95% CI	*p*	HB‐corrected *p*
Presence of interictal patterns				
Isolated discharges	.14	.01–2.79	.20	.40
RED type 1	.03	.00–.74	.032	.13
RED type 2	.09	.01–.90	.041	.12
Presence of ictal patterns				
Paroxysmal fast activity	.82	.11–6.30	.85	.85
Repetitive epileptiform discharges	.19	.03–1.07	.06	.30
Rhythmic slow activity	1.41	.25–7.99	.70	1.00
Suppression	.06	.00–1.73	.10	.41
None	.65	.08–5.10	.68	1.00

Abbreviations: CI, confidence interval; HB, Holm–Bonferroni; OR, odds ratio; RED, repetitive epileptiform discharges.

## 4 DISCUSSION

In this case–control study, we characterized interictal and seizure‐onset patterns on scalp EEG in patients with drug‐resistant focal epilepsy associated with MCD, compared to patients with other or no identifiable pathology for their focal epilepsy, and explored their potential association with seizure outcomes following resective epilepsy surgery. Among interictal patterns, RED type 1 and type 2 were significantly more prevalent in patients with MCDs. Additionally, focal fast epileptiform discharges were more frequently observed in MCDs after excluding individuals with nonlesional focal epilepsy. Regarding seizure‐onset patterns, paroxysmal fast activity was significantly associated with MCDs. Due to the small sample size, we were unable to reliably characterize EEG patterns across different MCD types. In the relatively small subset of patients who underwent epilepsy surgery, none of the identified interictal or seizure‐onset patterns demonstrated a significant correlation with postoperative seizure outcomes.

Consistent with established literature, isolated epileptiform discharges were the most common interictal pattern in both groups. RED type 1 and type 2 were significantly more prevalent in MCDs, which aligns with prior studies.^10^ These patterns likely reflect the high intrinsic epileptogenicity of the malformed cortical tissue, which is characterized by abnormal neuronal synchronization with increased excitatory and decreased inhibitory activity (γ‐aminobutyric acidergic [GABAergic] interneurons) compared to the adjacent normal tissue.^10^, ^24^ Increased excitability in MCDs also explains their association with focal fast epileptiform discharges,^25^, ^26^ which appeared to be more prevalent in MCDs after accounting for potential confounding from occult MCDs in the MRI‐negative controls. Variability in the excitation thresholds in the malformed cells may also contribute for this pattern.^25^ Although brushes are a pattern previously linked to FCD,^21^ they were absent in our MCD cohort, likely owing to the rarity of this EEG pattern and our relatively small sample size.

Paroxysmal fast activity at seizure onset was significantly more prevalent in patients with MCDs. This pattern has been previously reported in MCDs and correlates with low‐voltage fast activity (LVFA) observed in intracranial EEG.^12^, ^27^ Paroxysmal fast activity may reflect aberrant high‐frequency synchronization within the malformed tissue, where disrupted inhibitory interneurons and abnormal synaptic connectivity facilitate rapid ictal discharges.^28^ In dysplastic human neocortex tissue, LVFA‐like activity can be induced by 4‐aminopyridine injection, which enhances both glutamatergic and GABAergic transmission.^29^ Low‐voltage fast activity is thought to possibly result from increased inhibitory neuron firing, which in turn provokes an increase in excitatory neuron firing and seizure evolution.^30^

Although some variability in EEG patterns was noted across different MCD types, the majority of patterns were shared by all types. This potentially suggests overlapping mechanisms of epileptogenesis and ictogenesis among MCD types.^20^ Among ictal patterns, paroxysmal fast activity was not observed in patients with mild or complex MCDs. Mild MCDs may lack the architectural disruption needed to generate fast ictal discharges, whereas complex MCDs may exhibit a more diffuse epileptogenic zone. Paroxysmal fast activity tends to be observed in superficial epileptogenic focus.^12^ FCDs, in particular, are prone to paroxysmal and repetitive bursting activity due to hyperexcitability linked to enhanced glutamatergic interaction and reduced sensitivity to inhibition.^31^ The abnormal distribution of interneurons in the dysplastic cortex leads to decreased inhibitory postsynaptic current frequency and diminished cumulative GABA‐mediated synaptic currents.^32^ Rhythmic slow activity was most frequently observed in heterotopia, possibly reflecting slower propagation of ictal discharges, as this pattern is commonly associated with a deep epileptogenic focus.^12^

Postoperative seizure outcomes were favorable in most cases and controls undergoing resective epilepsy surgery, with no significant between‐group differences. The presence of RED type 1 and type 2 might be associated with unfavorable outcomes, although this association did not reach significance. Whereas prior studies have linked paroxysmal fast activity to better surgical outcomes compared to slower ictal rhythms,^12^, ^27^, ^33^, ^34^ in our cohort we did not observe this association. Intracranial studies have shown that LVFA pattern is frequently associated with favorable surgical outcomes, specifically when the epileptogenic zone is completely resected.^5^ Paroxysmal fast activity observed on scalp EEG may represent a noninvasive correlate of intracranial LVFA, highlighting the continuum between intracranial and scalp EEG patterns.^12^ It has been suggested that EEG electrodes recording higher frequency ictal discharges at seizure onset may be closer to the seizure onset zone, and thus predictive of favorable outcomes.^35^ Conversely, slower frequency activity at ictal onset may indicate a propagated electrographic pattern rather than a primary onset zone.^36^, ^37^

This study has limitations. First, the relatively small sample size of the MCD cohort, particularly within individual subtypes, may have limited our ability to detect subtle differences in EEG patterns across different MCD types or between MRI‐negative and MRI‐positive cases. Second, the low prevalence of certain EEG patterns (e.g., brushes and suppression) restricted our statistical power to assess their diagnostic specificity for MCDs. Third, the interrater reliability for infrequent EEG patterns (e.g., RED type 2, focal fast epileptiform discharges, and brushes) was poor to negligible. Although these EEG patterns are often used to help distinguish MCDs from other focal epilepsies, they should be interpreted cautiously and viewed as supportive rather than definitive diagnostic markers. Furthermore, the limited interrater reliability also reflects the inherent subjectivity of visual EEG interpretation. Future studies may benefit from incorporating quantitative or automated EEG analysis approaches to enhance consistency. Fourth, although we adjusted for ASM withdrawal, other potential confounders such as lesion extent and location were not systematically assessed. Fifth, undetected subtle MCDs on conventional MRI in the control group may have introduced heterogeneity. Future studies incorporating advanced imaging and postprocessing approaches are warranted. Sixth, VEM data for one control and one case were obtained after epilepsy surgery due to persistent seizures. This might have introduced confounding, as postoperative cortical reorganization and gliosis can alter the interictal and seizure‐onset patterns.^38^ Finally, in exploring the association between EEG patterns and postoperative seizure outcomes, we did not account for other prognostic factors such as the extent of resection, side of resection, history of febrile seizures, and neurodevelopmental comorbidities, which could influence postoperative seizure outcomes. Furthermore, given the small number of operated patients, the absence of significant association should not be interpreted as evidence that scalp EEG lacks prognostic utility.

## 5 CONCLUSIONS

MCDs exhibit distinct interictal and seizure‐onset scalp EEG patterns, including repetitive epileptiform discharges, focal fast epileptiform discharges, and paroxysmal fast activity, which may serve as supportive markers during presurgical evaluation. These EEG features should be interpreted as facilitating the differentiation of MCDs from other prevalent focal epilepsies, rather than as definitive electrophysiological signatures of MCD pathology. Although no significant associations between scalp EEG patterns and surgical outcomes were identified, these findings should be interpreted cautiously, given the limited number of operated patients and the potential influence of unmeasured surgical and pathological factors. Further research is warranted to characterize scalp EEG patterns across different MCD types and investigate their potential role in predicting surgical outcomes, guiding targeted high‐resolution MRI, and detecting MRI‐negative MCDs.

## AUTHOR CONTRIBUTIONS

**Lubna Shakhatreh:** Writing—original draft (lead); writing—review and editing (equal); conceptualization (equal); methodology (equal); investigation (lead); data curation (lead); formal analysis (equal); visualization (lead). **Liz Edenberg P. Quiles:** Investigation (supporting). **Zhibin Chen:** Writing—review and editing (equal); formal analysis (equal). **Ofer M. Gonen:** Investigation (supporting). **Saba Mohidat:** Investigation (supporting). **Lyn Millist:** Writing—review and editing (supporting); data curation (supporting). **Callum Hollis:** Data curation (supporting). **Andrea Sundram:** Data curation (supporting). **Piero Perucca:** Writing—review and editing (equal); conceptualization (equal); methodology (equal); supervision (equal). **Terence J. O'Brien:** Writing—review and editing (equal); conceptualization (equal); methodology (equal); supervision (equal). **Patrick Kwan:** Writing—review and editing (equal); conceptualization (equal); methodology (equal); supervision (equal).

## FUNDING INFORMATION

T.J.O. is supported by Investigator Grants from the National Health and Medical Research Council of Australia (APP1176426 and APP2034258). L.S. received support from the Monash International Tuition Scholarship.

## CONFLICT OF INTEREST STATEMENT

P.P. is supported by an Emerging Leadership Investigator Grant from the National Health and Medical Research Council (NHMRC; APP2017651), the University of Melbourne, Monash University, and the Austin Medical Research Foundation. He has received speaker honoraria or consultancy fees to his institution from Chiesi, Eisai, Jazz Pharmaceuticals, LivaNova, Novartis, Sun Pharma, Supernus, and UCB Pharma, outside of the submitted work. He is on the board of the International Registry of Antiepileptic Drugs and Pregnancy, a nonprofit organization that has received financial support from Accord, Angelini, Bial, EcuPharma, Eisai, Glenmark, GW Pharma, GlaxoSmithKline, Sanofi, SF Group, Teva, UCB, and Zentiva. He is Deputy Editor for *Epilepsia Open*. T.J.O. is supported by Investigator Grants from the NHMRC (APP1176426 and APP2034258). He reports research support and consulting fees to his institution from Eisai, UCB Pharma, LivaNova, Bright Minds Biopharma, Kinoxis Pharmaceuticals, Jazz Pharmaceuticals, and Supernus outside of the submitted work. P.K. is supported by an NHMRC Investigator Grant (GNT2025849). He/his institution has received research grants and consultancy fees from Angelini, Eisai, Jazz Pharmaceuticals, UCB Pharma, and LivaNova. The other authors have no conflicts of interest. We confirm that we have read the Journal's position on issues involved in ethical publication and affirm that this report is consistent with those guidelines.

## Supporting information


Data S1.

## Data Availability

All relevant data supporting the findings of this study are included within the article and its Supporting Information. Requests for access to additional anonymized data may be requested from the corresponding author (L.S.) in line with ethics approval.
