# Supplementary material for: Interictal and seizure‐onset scalp electroencephalographic patterns in malformations of cortical development
Source: Epilepsia. 2026 Mar 14;67(6):2979–91. doi: 10.1002/epi.70193 (PMC13285233; doi:10.1002/epi.70193)
Supplement: Supplementary file 1 — Data S1. [file EPI-67-2979-s001.docx]

**Supplementary tables:**

**Supplementary Table 1:** The framework used to classify the described scalp EEG patterns in this study.

| **Interictal EEG patterns** | |
| --- | --- |
| **1** | **Repetitive epileptiform discharges (RED) type 1:** trains of repetitive, rhythmic 4-10 Hz sharp waves or spikes lasting 1-4 sec.^5^ |
| **2** | **Repetitive epileptiform discharges (RED) type 2:** quasi-continuous, slower, rhythmic 2-7 Hz sharp wave activity.^5^ |
| **3** | **Brushes:** sub-continuous rhythmic spikes and polyspikes and waves, usually at a frequency of between 1 and 3 Hz, alternating with short bursts of fast discharges and interrupted by suppression of electrical activity.^17^ |
| **4** | **Focal fast epileptiform discharges:** focal spikes or polyspikes with a frequency of at least 10 Hz lasting at least 3 seconds.^6^ |
| **5** | **Isolated discharges**: either spikes, spike-and-waves or polyspikes.^6^ |
| **Seizure-onset EEG patterns** | |
| **1** | **Paroxysmal fast activity:** sinusoidal activity >= 13 Hz.^7^ |
| **2** | **Rhythmic slow activity:** sinusoidal activity <13 Hz.^7^ |
| **3** | **Repetitive epileptiform discharges:** Spike-and-wave or sharp-and-wave activity or repetitive spikes at <13 Hz.^7^ |
| **4** | **Focal fast wave:** rhythmic slow wave superimposed with fast activity.^18^ |
| **5** | **Suppression:** suppression of background activity to <= 10 microV.^7^ |

**Supplementary Table 2:** Differences in clinical and VEM characteristics between cases and controls

| **Clinical and VEM characteristics** | **MCD vs Controls** | **MCD vs Controls (excluding non-lesional)** |
| --- | --- | --- |
|  | **p-value** | **p-value** |
| Female sex (n, %) | 0.40 | 0.34 |
| Median age at seizure onset in years (IQR) | 0.009 | 0.011 |
| Median age at VEM admission in years (IQR) | 0.017 | 0.004 |
| Median number of ASMs at time of VEM admission (IQR) | 0.034 | 0.063 |
| Changes in ASMs made during VEM (n; %)^a^ | 0.061 | 0.074 |
| Number of VEM days (range) | 0.44 | 0.25 |
| Median non-convulsive seizure frequency score at time of VEM admission | 0.80 | 0.77 |
| Median convulsive seizure frequency score at time of VEM admission | 0.46 | 0.33 |

a: changes in ASMs were unknown in 3 controls.

**Supplementary Table 3:** Number of events marked and inter-rater reliability for EEG patterns between the two raters.

|  | **Reviewer 1** | **Reviewer 2** | **ICC(*A*,1)** | **(95% CI)** |
| --- | --- | --- | --- | --- |
| Total number of seizures | 348 | 393 | 0.964 | (0.929-0.982) |
| Interictal patterns |  |  |  |  |
| Focal fast epileptiform discharges | 0 | 3 | 0.000 | (-0.323-0.328) |
| Isolated discharges | 1144 | 1061 | 0.987 | (0.974-0.993) |
| RED type 1 | 73 | 54 | 0.754 | (0.561-0.869) |
| RED type 2 | 19 | 28 | -0.009 | (-0.353-0.331) |
| Brushes | 6 | 0 | 0.000 | (-0.334-0.334) |
| Seizure-onset patterns |  |  |  |  |
| Paroxysmal fast activity | 88 | 90 | 0.941 | (0.886-0.97) |
| Repetitive epileptiform discharges | 94 | 84 | 0.983 | (0.967-0.992) |
| Rhythmic slow activity | 143 | 169 | 0.924 | (0.854-0.961) |
| Suppression | 2 | 15 | -0.042 | (-0.335-0.276) |

CI: confidence interval; ICC(*A*,1): intraclass correlation coefficient for absolute agreement.

**Supplementary Table 4:** Interictal and seizure-onset EEG patterns across MCDs and controls, adjusted for ASM withdrawal during VEM.

|  | **MCDs vs Controls** | | | | **MCD vs Controls (excl. non-lesional)** | | | |
| --- | --- | --- | --- | --- | --- | --- | --- | --- |
|  | **OR/RoM** | **(95% CI)** | **p-value** | **HB-corrected p-value** | **OR/RoM** | **(95% CI)** | **p-value** | **HB-corrected p-value** |
| Presence of interictal patterns | | | | | | | | |
| Focal fast epileptiform discharges | 5.23 | (1.04-26.2) | 0.044 | 0.18 | 15.4 | (0.79-299) | 0.071 | 0.28 |
| Isolated discharges | 1.16 | (0.46-2.90) | 0.75 | 1.00 | 1.11 | (0.41-2.96) | 0.84 | 1.00 |
| RED type 1 | 6.21 | (2.31-16.7) | <0.001 | 0.002 | 7.31 | (2.24-23.9) | 0.001 | 0.006 |
| RED type 2 | 4.07 | (1.49-11.1) | 0.006 | 0.031 | 4.69 | (1.40-15.7) | 0.012 | 0.060 |
| Brushes | 0.98 | (0.04-26.7) | 0.99 | 0.99 | 1.44 | (0.02-105) | 0.87 | 0.87 |
| Presence of seizure-onset patterns | | | | | | | | |
| Paroxysmal fast activity | 9.76 | (3.29-28.9) | <0.001 | <0.001 | 9.46 | (2.67-33.5) | <0.001 | 0.002 |
| Repetitive epileptiform discharges | 2.50 | (1.15-5.44) | 0.021 | 0.084 | 3.07 | (1.30-7.29) | 0.011 | 0.043 |
| Rhythmic slow activity | 0.82 | (0.39-1.72) | 0.596 | 1.00 | 0.80 | (0.36-1.77) | 0.58 | 1.00 |
| Suppression | 0.77 | (0.12-4.98) | 0.787 | 0.79 | 0.71 | (0.10-5.23) | 0.74 | 0.74 |
| None | 0.62 | (0.24-1.60) | 0.326 | 0.98 | 0.55 | (0.20-1.50) | 0.24 | 0.72 |
|  |  |  |  |  |  |  |  |  |

CI: confidence interval; HB: Holm-Bonferroni; HS: hippocampal sclerosis; MCDs: malformations of cortical development; OR: odds ratio; RoM: ratio of means.

**Supplementary Table 5:** Epilepsy surgery outcomes across MCDs and controls.

|  | **MCD** | **Controls** | | | | **MCD vs Controls** | **MCD vs Controls (excl. non-lesional)** |
| --- | --- | --- | --- | --- | --- | --- | --- |
|  |  | **HS** | **Acquired Epilepsy** | **Non-lesional** | **Subtotal** |  |  |
|  |  |  |  |  |  | **p-value** | **p-value** |
| N | 10 | 18 | 6 | 4 | 28 |  |  |
| Surgical outcomes (N, %) |  |  |  |  |  | 0.65 | 0.63 |
| Unfavourable | 3 (20.0) | 4 (22.2) | 0 (0) | 2 (50.0) | 7 (25.0) |  |  |
| Favourable | 7 (70.0) | 14 (77.8) | 6 (100.0) | 2 (50.0) | 21 (75.0) |  |  |

HS: hippocampal sclerosis; MCDs: malformations of cortical development.
